# PRDM1 Knockdown Promotes Ferroptosis and Sunitinib Sensitivity by Modulating the PI3K/Akt Signaling Through Inhibition of ESM1 Transcription in Renal Cell Carcinoma

**DOI:** 10.1002/kjm2.70187

**Published:** 2026-02-16

**Authors:** Yi‐Shuai Zhang, Xin‐Qi Pei, Ji‐Ping Sun, Chen‐Hui Ma, Xin‐Yang Wang, Xu‐Dong Li

**Affiliations:** ^1^ Department of Urology The First Affiliated Hospital of Xi'an Jiaotong University Xi'an China; ^2^ Department of Nephrology The First Affiliated Hospital of Xi'an Jiaotong University Xi'an China

**Keywords:** ESM1, PI3K/Akt signaling, PRDM1, renal cell carcinoma (RCC), sunitinib sensitivity

## Abstract

Renal cell carcinoma (RCC) is a commonly occurring and considerable burden on public health. Although chemotherapy benefits patients with advanced RCC, sunitinib resistance leads to a poor prognosis. Ferroptosis is also involved in sunitinib resistance in RCC. PR domain‐containing protein 1 with a zinc finger domain (PRDM1) is a tumor regulator in many types of cancer. However, its biological function in RCC has not been reported. This study aimed to investigate the role of PRDM1 in ferroptosis and sunitinib resistance in RCC and explore the underlying mechanisms. Bioinformatics analysis was performed to analyze the expression of related genes in RCC. PRDM1‐silencing and overexpressing cells were constructed to confirm the role of PRDM1 in RCC in vitro. Our results showed that PRDM1 expression was markedly upregulated in RCC tissues and cell lines. PRDM1 knockdown significantly induced ferroptosis in RCC cells. Furthermore, knockdown of PRDM1 elevated the sensitivity of RCC cell lines to Mechanistically, PRDM1 directly bound to ESM1 and regulated its transcription. Subsequently, ESM1 overexpression reversed the effects of si‐PRDM1 on ferroptosis and sunitinib sensitivity in RCC cells, and these effects were mitigated by a PI3K inhibitor. Finally, PRDM knockdown exhibited anti‐tumor effects in a xenograft animal model. Taken together, our study shows that PRDM1 silencing promotes ferroptosis and sunitinib sensitivity by inhibiting ESM1 transcription and modulating PI3K/Akt signaling in RCC. Our findings thus provide novel insights for therapeutically targeting RCC.

## 1 Introduction

Renal cell carcinoma (RCC) is a commonly occurring and considerable burden on public health. Over the past 10 years, the incidence of RCC is increasing worldwide [1]. Based on the USA statistics, approximate 82,000 new diagnoses and 15,000 mortality cases are predicted in 2023 [2]. Among the various subtypes, clear cell renal cell carcinoma (ccRCC) remains the most common type and has been considered as a highly aggressive urological malignancy with a significant mortality rate [3]. Currently, therapeutic approaches for advanced ccRCC are limited, and its 5‐year survival rate remains unsatisfactory [4]. Discovery of novel biomarkers and therapeutic targets is necessary for early diagnosis, treatment, and prognostic assessment of ccRCC [4].

Sunitinib is one of the main antitumor drugs for the treatment of metastatic RCC [5]. However, more than 20% of patients with advanced RCC exhibit drug resistance, and most of them will eventually develop sunitinib resistance [6]. Sunitinib is a commonly used multi‐kinase inhibitory drug that inhibits a number of receptor tyrosine kinases such as platelet‐derived growth factor receptor (PDGFR) and vascular endothelial growth factor receptor (VEGFR) [7]. In addition to inhibiting kinases, sunitinib also induces ferroptosis, which is a distinct cell death paradigm driven by lipid peroxide accumulation [8, 9]. Importantly, recent research has demonstrated that inducers or activators of ferroptosis contribute to overcoming sunitinib resistance in RCC [10, 11]. It is crucial to ascertain mechanisms underlying sunitinib resistance, which will contribute to overcoming it and promote the development of therapeutic strategies in RCC.

PR domain‐containing protein 1 with zinc finger domain (PRDM1), also known as Blimp‐1, is an important transcription factor [12]. PRDM1 is a DNA‐binding protein that binds to target sequences of specific genes and thus activates or inhibits their transcription, thereby regulating cell proliferation, apoptosis, pyroptosis, and immune responses in various diseases [12, 13]. PRDM1 plays critical roles in the development of many human diseases including cancers [14]. PRDM1 is reportedly involved in tumor development of diverse cancers including hepatocellular carcinoma [15], lymphoid tumors [14], thyroid cancer [16], and pancreatic adenocarcinoma (PAAD) [17]. These studies also proved that PRDM1 is implicated in many processes associated with tumor progression, such as cell differentiation [14], cancer immune evasion [15, 16], ferroptosis [16], and resistance [18]. However, the potential functions of PRDM1 in RCC remain unclear. Therefore, this study aimed to investigate the role of PRDM1 in ferroptosis and sunitinib resistance in RCC and explore the underlying mechanisms.

## 2 Methods and Materials

### 2.1 Datasets Source and Analysis

The GSE53757 and GSE40435 gene expression profile datasets were obtained from the GEO database (https://www.ncbi.nlm.nih.gov/geo). PRDM1‐related factors in renal clear cell carcinoma (KIRC) were obtained from Linkedomics (https://www.linkedomics.org/login.php; *p* < 0.05; cor. *R* ≥ 0.5). PRDM1 targets were selected using KnockTF 2.0 (http://www.licpathway.net/KnockTF/, log2FC ≤ −1). The genes co‐expressed with ESM1 were analyzed using LinkedOmics (http://www.linkedomics.org/login.php; *p* < 0.05).

### 2.2 Cell Culture and Transfections

Normal human renal tubular epithelial cells (HK‐2 cells) and RCC cell lines (A498, 786‐O, and ACHN) were obtained from the Cell Bank of the Type Culture Collection of the Chinese Academy of Sciences (Shanghai, China). The cells were cultured in Dulbecco's modified Eagle's medium (DMEM; Cat. No. 11885084; Gibco, Grand Island, NY, USA) supplemented with 10% fetal bovine serum (FBS; Cat. No. A5256701; Gibco). Cells were maintained in a humidified incubator with 5% CO_2_ at 37°C. These cell lines were tested with no mycoplasma contamination, and cells of passage 6 to 8 were used.

The 786‐O and ACHN cells were transfected with si‐PRDM1#1 or si‐PRDM1#2 (Cat. No. A01001; Shanghai GenePharma, Shanghai, China) to knockdown PRDM1. In addition, pcDNA/PRDM1 or pcDNA/ESM1 was transfected into 786‐O and ACHN cells using Lipofectamine 2000 (Cat. No. 11668019; Invitrogen; Thermo Fisher Scientific, Waltham, MA, USA) to upregulate the expression of PRDM1 or ESM1. At 72 h post‐transfection, the expression levels of PRDM1 and ESM1 were measured by western blot analysis. To inhibit ferroptosis, cells were treated with 1 μM Ferrostatin‐1 (Fer‐1).

### 2.3 Real‐Time Quantitative PCR (RT‐qPCR)

Total RNA samples from the cell lines were extracted using TRIzol reagent (Cat. No. 15596026CN; Invitrogen) prior to the determination of RNA purity and quantity. RNA samples were reverse‐transcribed into cDNA using a reverse transcription kit (Cat. No. RK20400; ABclonal Technology, Wuhan, China). RT‐qPCR was conducted on a LightCycler 480 Instrument II (Roche Applied Science, Mannheim, Germany) using Brilliant SYBR Green Master Mix (Cat. No. 172‐5120; Bio‐Rad Laboratories, Hercules, CA, USA). The primer sequences used were as follows: PRDM1 (forward: 5′‐AAGCAACTGGATGCGCTATGT‐3′, reverse: 5′‐GGGATGGGCTTAATGGTGTAGAA‐3′), ESM1 (forward: 5′‐ TTGCTACCGCACAGTCTCAG‐3′, reverse: 5′‐GCAGGTCTCTCTGCAATCCA‐3′), and GAPDH (forward: 5′‐CTTAGTTGCGTTACACCCTTTCTTG‐3′, reverse: 5′‐CTGTCACCTTCACCGTTCCAGTTT‐3′). The PRDM1 and ESM1 mRNA expression levels were normalized to GAPDH and expressed as fold change of the control group.

### 2.4 Western Blot

Total proteins from cell lines and tumor tissues were extracted using radioimmunoprecipitation assay (RIPA) buffer (Cat. No. P0013B; Beyotime Biotechnology, Shanghai, China). After determining the protein concentration, protein samples were subjected to electrophoresis on 10% sodium dodecyl sulfate (SDS)‐sulfate‐polyacrylamide gels. The proteins were then transferred to the PVDF membrane, which was subsequently blocked with a blocking buffer (Cat. No. P0023B; Beyotime Biotechnology). The membrane was then incubated with primary antibodies against PRDM1 (Cat. No. 9115; Cell Signaling Technology, Boston, MA, USA), glutathione peroxidase 4 (GPX4; Cat. No. 52455; Cell Signaling Technology), octamer‐binding transcription factor 4 (OCT4; Cat. No. 2750; Cell Signaling Technology), SRY (sex‐determining region Y)‐box 2 (SOX2; Cat. No. 2748; Cell Signaling Technology), ESM1 (Cat. No. AB103590; Abcam, Cambridge, MA, USA), p‐PI3K (Cat. No. ab138364; Abcam), PI3K (Cat. No. ab131067; Abcam), p‐Akt (Cat. No. ab38449; Abcam), Akt (Cat. No. ab8805; Abcam), β‐actin (Cat. No. ab8227; Abcam), and the corresponding secondary antibodies (Cat. No. ab6721; Abcam). Finally, the membranes were immersed in enhanced chemiluminescence reagent (Cat. No. 32106; Pierce, Rockford, IL, USA) to observe the protein bands. The protein levels were normalized to β‐actin and expressed as fold change of the control group.

### 2.5 Associated Indicators Measurement

The levels of lactate dehydrogenase (LDH), malondialdehyde (MDA), and Fe^2+^ were determined using LDH Cytotoxicity Assay Kits (Cat. No. C0016; Beyotime Biotechnology), MDA Assay Kits (Cat. No. S0131S; Beyotime Biotechnology), and ferrous iron colorimetric assay kits (Cat. No. E‐BC‐K773‐M; Elabscience, Wuhan, China). Reactive oxygen species (ROS) production was detected using a ROS Assay Kit with CM‐H_2_DCFDA (Cat. No. S0035S; Beyotime Biotechnology). Finally, intracellular fluorescence was analyzed using flow cytometry (Becton Dickinson, San Jose, CA, USA).

### 2.6 Cell Proliferation Assay

Cells (2 × 10^4^) were seeded into 12‐well plates and subjected to different treatments and transfection for 48 h. After that, 10 μL CCK‐8 reagent from Cell Counting Kit‐8 (Cat. No. C0037; Beyotime Biotechnology) was added to the cells for 2 h. Finally, optical density (OD) values were determined on a microplate spectrophotometer (BioTek Instruments, Winooski, VT, USA) based on absorbance at 450 nm.

### 2.7 Colony Formation Assay

Following different treatments, 2000 cells/well were seeded in 6‐well plates. Two weeks after seeding, the colonies were fixed with 4% cell fixative and stained with 0.1% crystal violet (Cat. No. Y268090; Beyotime Biotechnology) for 10 min at room temperature. Finally, all colonies containing more than 50 cells were counted.

### 2.8 Luciferase Reporter Assay

Based on the motif of PRDM1 and the binding sites of the promoter region of ESM1, a wild‐type/mutant‐type ESM1 luciferase reporter plasmid (pGL3‐basic plasmid) was constructed. A luciferase reporter assay was conducted by co‐transfection with si‐PRDM1#2/si‐con, wild‐type/mutant‐type pGL3‐ESM1 plasmid, and the Renilla luciferase reporter vector. After 24 h, cells were collected to determine luciferase activity using a Dual‐Luciferase Reporter Assay System (Cat. No. E1910; Promega, Madison, WI, USA).

### 2.9 Chromatin Immunoprecipitation (ChIP) Assay

The ChIP assay was carried out using the Pierce Agarose ChIP Kit (Cat. No. 26156; Pierce), following the manufacturer's protocol. Briefly, cells (1 × 10^7^ cells) were fixed with 1% formaldehyde and lysed in a lysis buffer. The samples were then sonicated in an ultrasonic processor with 10 cycles of 6 s power‐on and 10 s power‐off interval. Antibodies against PRDM1 (anti‐PRDM1) or negative control IgG were used for subsequent immunoprecipitation. Finally, the chromatin protein/DNA complexes were eluted and the target DNA fragments were purified from the elution and subjected to RT‐PCR.

### 2.10 Animal Experiments

Thirty 6‐week‐old female nude mice (BALB/c nu/nu; Experimental Animal Center, Xi'an Jiaotong University, Xi'an, China) were used for in vivo animal experiments. All experimental protocols were approved by the Animal Care and Use Committee of the First Affiliated Hospital of Xi'an Jiaotong University (Xi'an, China). ACHN cells were transfected with a lentivirus carrying a specific shRNA targeting PRDM1 (sh‐PRDM1) or a negative control shRNA (sh‐con). Then 3 × 10^6^ sh‐PRDM1‐/sh‐con‐transfected ACHN cells were subcutaneously inoculated into the right flanks of the nude mice (*n* = 5). Tumor volumes were measured every 7 days. Five weeks after tumor inoculation, the xenografts were isolated and weighed. The volume of the xenograft was examined using a digital caliper and calculated using the formula 0.5 × length × width^2^.

### 2.11 Histopathology Analysis Methods

The xenografts were harvested, fixed with 4% paraformaldehyde, and finally embedded in paraffin and sectioned into 5‐μm sections for histopathological examination. For hematoxylin and eosin (H&E) staining, tissue sections were deparaffinized and stained with H&E (Cat. No. G1120; Solarbio, Beijing, China). For the immunohistochemistry (IHC) assay, tissue sections were deparaffinized, rehydrated, blocked, and incubated with a primary antibody against Ki‐67 (Cat. No. 34330; Cell Signaling Technology) and a secondary antibody (Cat. No. ab6721; Abcam). Finally, 3, 3′‐diaminobenzidine (DAB) substrate solution (Cat. No. P0202; Beyotime Biotechnology) was added to the sections to visualize the immunoreaction.

### 2.12 Statistical Analysis

Statistical analysis was performed using SPSS software version 17.0 (SPSS Inc., Chicago, IL, USA). One‐way analysis of variance or Student's *t*‐test was conducted to analyze differences among multiple groups or between two groups. Experiments were performed with 3 biological replicates × 3 technical replicates, unless otherwise indicated. Data were expressed as means ± standard error of the mean (SEM). Data were analyzed using Student's *t*‐test. The log‐rank test and Kaplan–Meier analysis were conducted to determine overall survival and recurrence. *p*‐values were two‐sided, and *p* < 0.05 indicated a statistically significant difference.

## 3 Results

### 3.1 Increased Expression of PRDM1 in RCC

**FIGURE 1:**
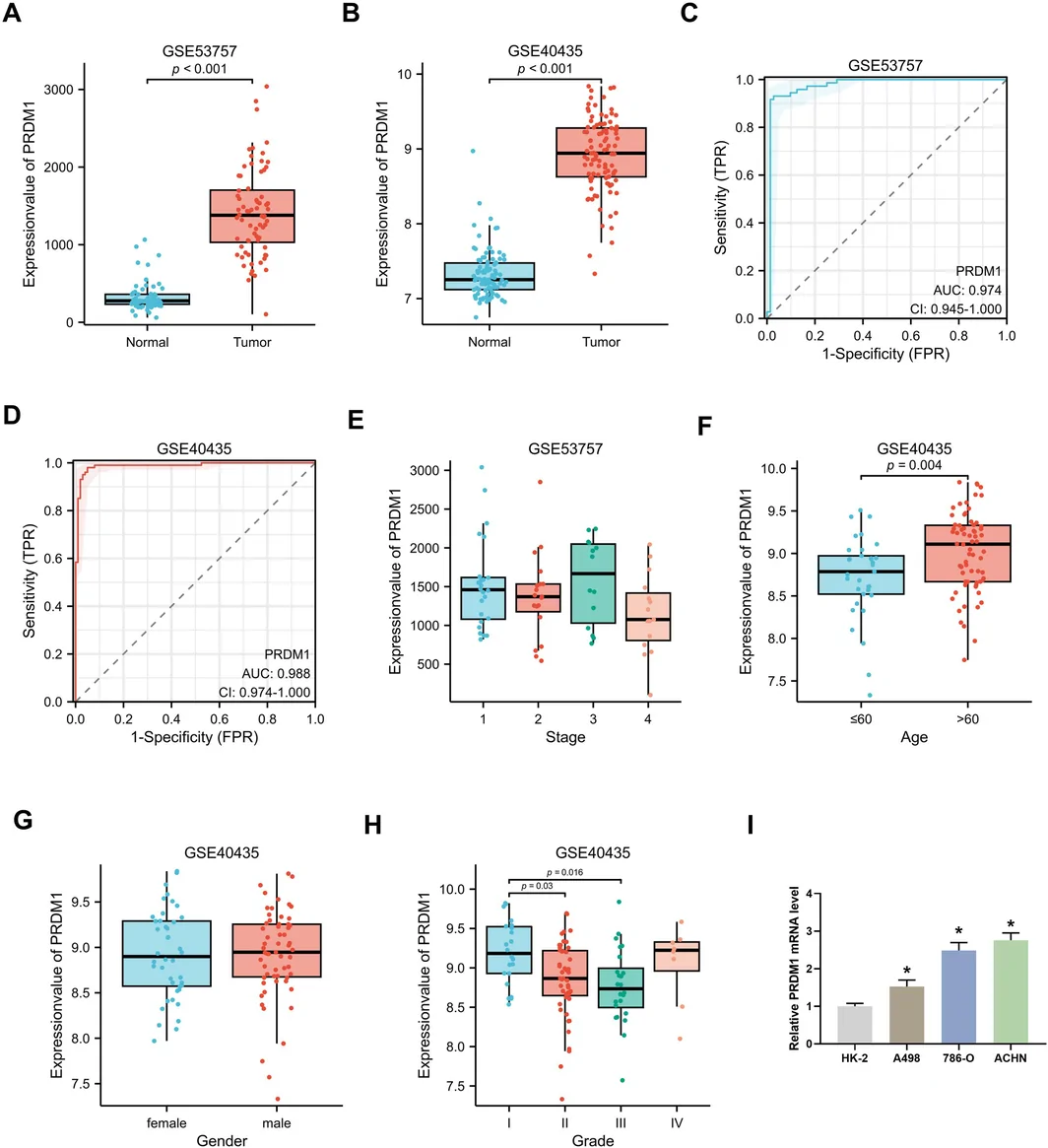
PRDM1 was highly expressed in RCC. (A, B) The expression levels of PRDM1 were markedly upregulated in RCC tissues from datasets GSE53757 and GSE40435. (C, D) ROC curves showing the diagnostic value of PRDM1 in RCC. (E–H) Different expression of PRDM1 in RCC patients with different stage, age, gender, and grade. (I) PRDM1 was highly expressed in RCC cell lines including A498, 786‐O, and ACHN cells. *p < 0.05 versus HK‐2 cells.

Based on the analysis of GSE53757 and GSE40435, the expression levels of PRDM1 were markedly upregulated in RCC tissues compared to that in normal controls (Figure 1A,B). ROC curves showed the diagnostic value of PRDM1 in RCC (AUC = 0.974 and 0.988, respectively) (Figure 1C,D). Moreover, we found significant differences in PRDM1 expression between patients aged ≤ 60 years and those aged > 60 years, as well as among patients with different grades (Figure 1E–H). Next, we evaluated the expression levels of PRDM1 in different RCC cell lines. RT‐PCR results indicated that PRDM1 was highly expressed in A498, 786‐O, and ACHN cells compared to that in control HK‐2 cells (Figure 1I).

### 3.2 Knockdown of PRDM1 Promoted Ferroptosis in RCC Cells

**FIGURE 2:**
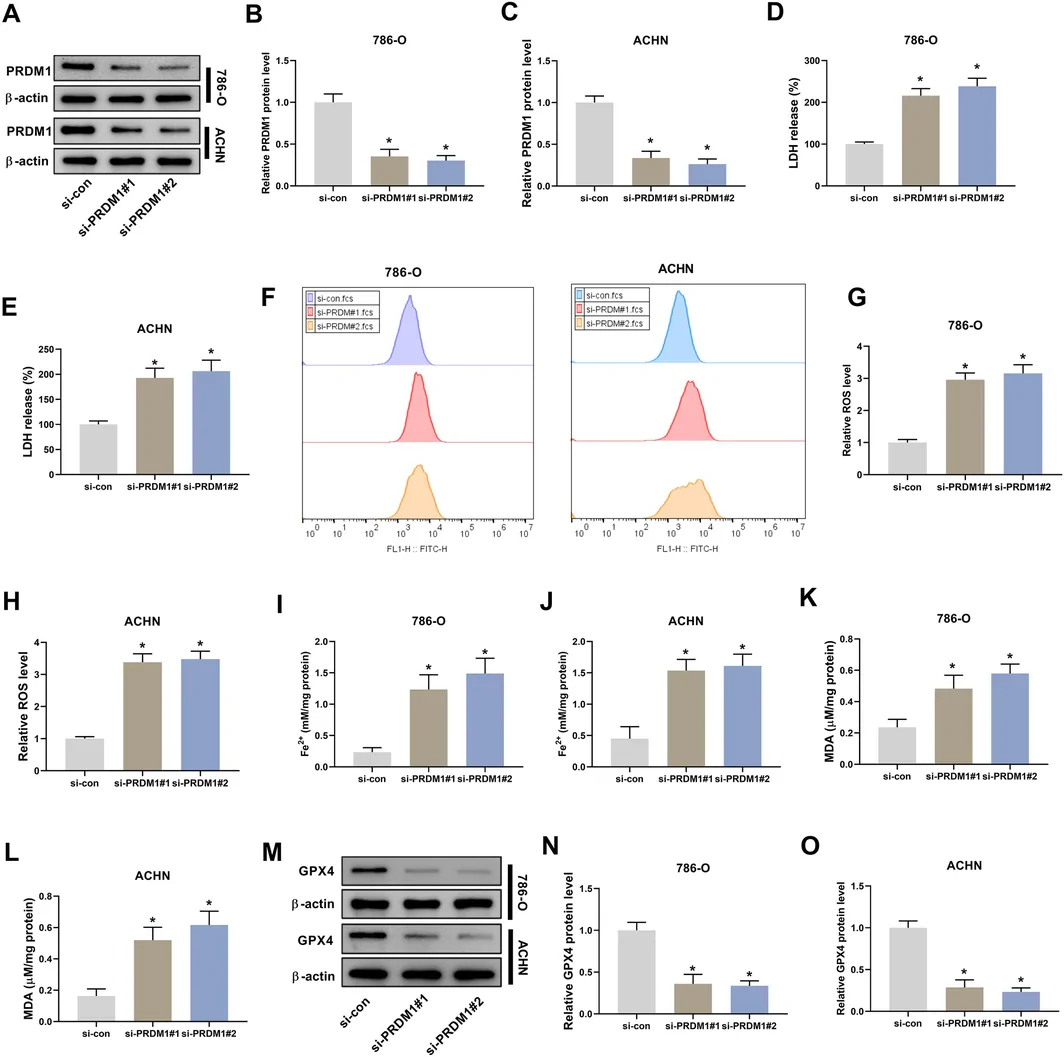
Knockdown of PRDM1 promoted ferroptosis in RCC cell lines. (A–C) Western blot for detecting the PRDM1 expression level after transfection with si‐PRDM1#1 or si‐PRDM1#2 in 786‐O and ACHN cells, respectively. (D, E) LDH levels in cells with different transfections. (F–H) Flow cytometry for ROS production. (I–L) Changes in Fe^2+^ level and MDA content after transfection with si‐PRDM1#1 or si‐PRDM1#2 in 786‐O and ACHN cells, respectively. (M–O) Western blot analysis for determination of GPX4 expression in 786‐O and ACHN cells. *p < 0.05 versus cells transfected with si‐con.

To investigate the role of PRDM1 in ferroptosis in RCC cells, si‐PRDM1#1 or si‐PRDM1#2 was transfected into 786‐O and ACHN cells. Based on the results of western blotting, PRDM1 expression levels dramatically decreased in both si‐PRDM1#1‐ and si‐PRDM1#2‐transfected cell lines (Figure 2A–C). In addition, LDH assay results showed that LDH levels significantly increased after transfection with si‐PRDM1#1 or si‐PRDM1#2 (Figure 2D,E). PRDM1 knockdown also caused a significant increase in ROS production, Fe^2+^ levels, and MDA content (Figure 2F–L). Furthermore, we observed that GPX4 expression decreased in si‐PRDM1#1‐ or si‐PRDM1#2‐transfected cells (Figure 2M–O). Moreover, addition of Fer‐1 attenuated the effects of PRDM1 silencing on these processes (Figure S1). These results indicate that PRDM1 knockdown promotes ferroptosis in RCC cells.

### 3.3 Knockdown of PRDM1 Enhanced Sunitinib Sensitivity via Stemness Regulation

**FIGURE 3:**
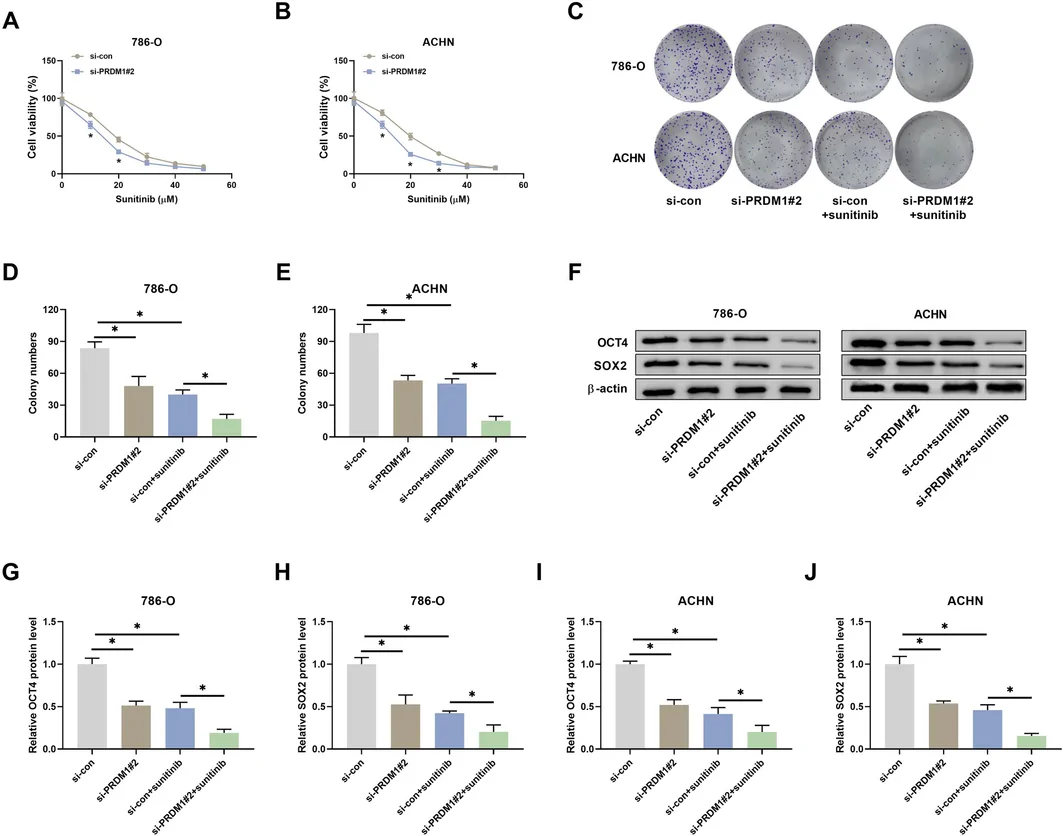
Knockdown of PRDM1 enhanced the sunitinib sensitivity via stemness regulation. RCC cells were transfected with si‐con or si‐PRDM1#2 for 72 h. Then, cells were treated with a series of concentrations of sunitinib for 24 h. (A, B) Cells viability was measured by CCK‐8 assay. (C–E) Colony formation assay was used to detect cell proliferation. (F–J) Western blot for detecting the expression levels of stemness‐associated markers OCT4 and SOX2. *p < 0.05 between two groups.

Next, we examined the role of PRDM1 in sunitinib sensitivity of RCC cells. As shown in Figure 3A,B, knockdown of PRDM1 enhanced sunitinib‐induced loss of viability in 786‐O and ACHN cells. Additionally, PRDM1 knockdown caused a significant reduction in colony number and enhanced the inhibitory effect of sunitinib on colony formation (Figure 3C–E). Because cancer stemness is the determinant factor for chemoresistance in RCC, we investigated the effect of PRDM1 on RCC cell stemness. Western blot was performed to test the expression of stemness‐associated markers, OCT4 and SOX2, in RCC cells. The results showed that the expression levels of OCT4 and SOX2 markedly decreased in si‐PRDM1#2 transfected cells. Transfection with si‐PRDM1#2 also reversed the decrease in OCT4 and SOX2 expression in sunitinib‐treated RCC cells (Figure 3F–J).

### 3.4 PRDM1 Directly Targeted ESM1

**FIGURE 4:**
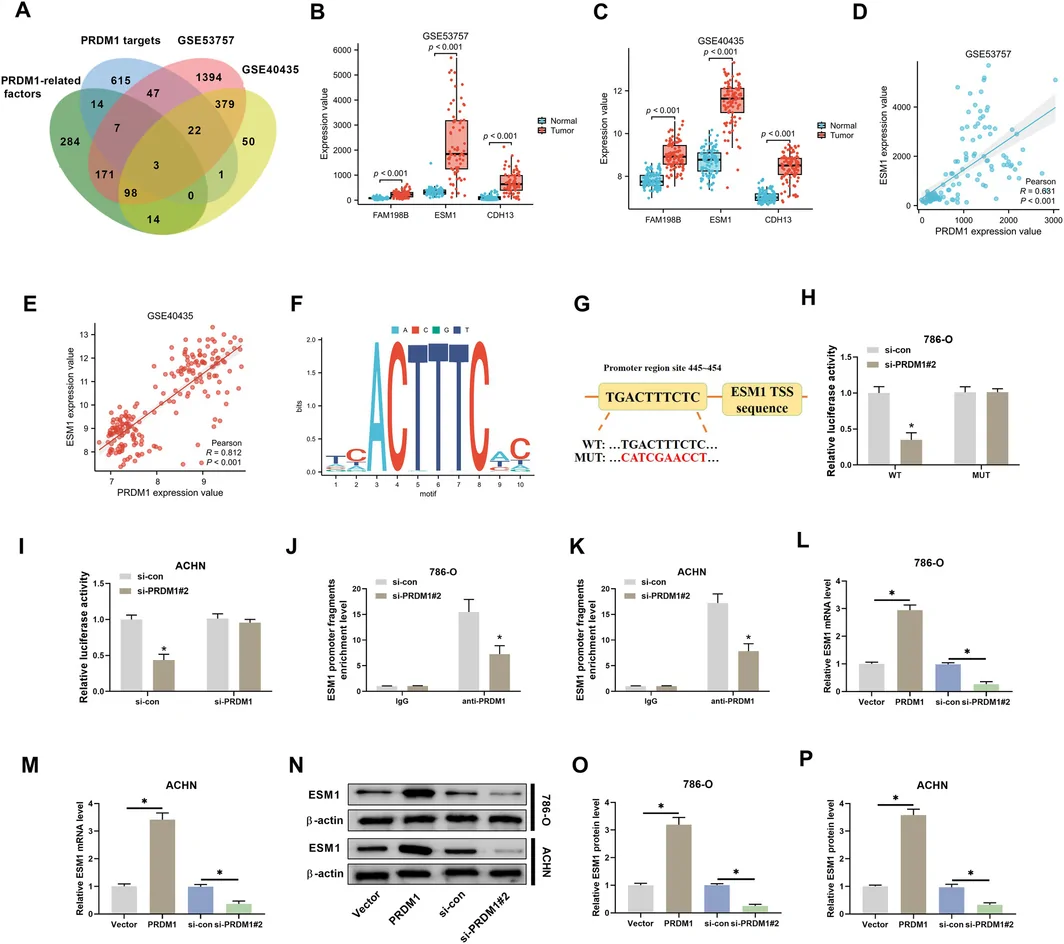
PRDM1 directly targeted ESM1 and regulated its transcription. (A) Venn analysis for GSE53757, GSE40435, PRDM1 targets dataset, and PRDM1‐related factors dataset. (B, C) Expression levels of FAM198B, ESM1, and CDH13 in RCC tissues from datasets GSE53757 and GSE40435. (D, E) Pearson correlation analysis for the association between ESM1 and PRDM1 expression in RCC. (F–I) Based on the motif of PRDM1 and binding sites of promoter region of ESM1 (F), wild‐type and mutant‐type ESM1 luciferase reporter plasmids were constructed (G), and then luciferase reporter assay was performed (H, I). (J, K) ChIP assay for the interaction between ESM1 and PRDM1. (L–P) RT‐qPCR and western blot for detecting the regulatory effect of PRDM1 on ESM1 expression. *p < 0.05 between two groups.

To identify potential downstream targets of PRDM1, the GSE53757, GSE40435, PRDM1 targets dataset, and PRDM1‐related factors dataset were obtained. Venn analysis identified three intersected genes (*FAM198B, ESM1*, and *CDH13*) (Figure 4A). Among these three genes, ESM1 was the most differentially expressed in RCC; thus, it was selected for further analysis (Figure 4B,C). Pearson's correlation analysis showed that ESM1 expression was closely associated with PRDM1 expression in RCC (Figure 4D,E). Next, the motifs of PRDM1 and the binding sites in the promoter region of ESM1 were predicted and are presented in Figure 4F. Further, luciferase reporter assays proved that cells co‐transfected with si‐PRDM1#2 and wild‐type ESM1 luciferase reporter plasmid exhibited an obvious decrease in luciferase activity (Figure 4G–I). The CHIP results in Figure 4J,K showed that PRDM1 was correlated with the ESM1 promoter. In addition, the mRNA and protein levels of PRDM1 were markedly upregulated in PRDM1‐overexpressing cells, but downregulated in PRDM1‐silencing cells (Figure 4L–P). These results indicated that PRDM1 directly binds to ESM1 and regulates its transcription.

### 3.5 PRDM1 Regulates the PI3K/Akt Pathway Through Mediation of ESM1 Expression in RCC Cells

**FIGURE 5:**
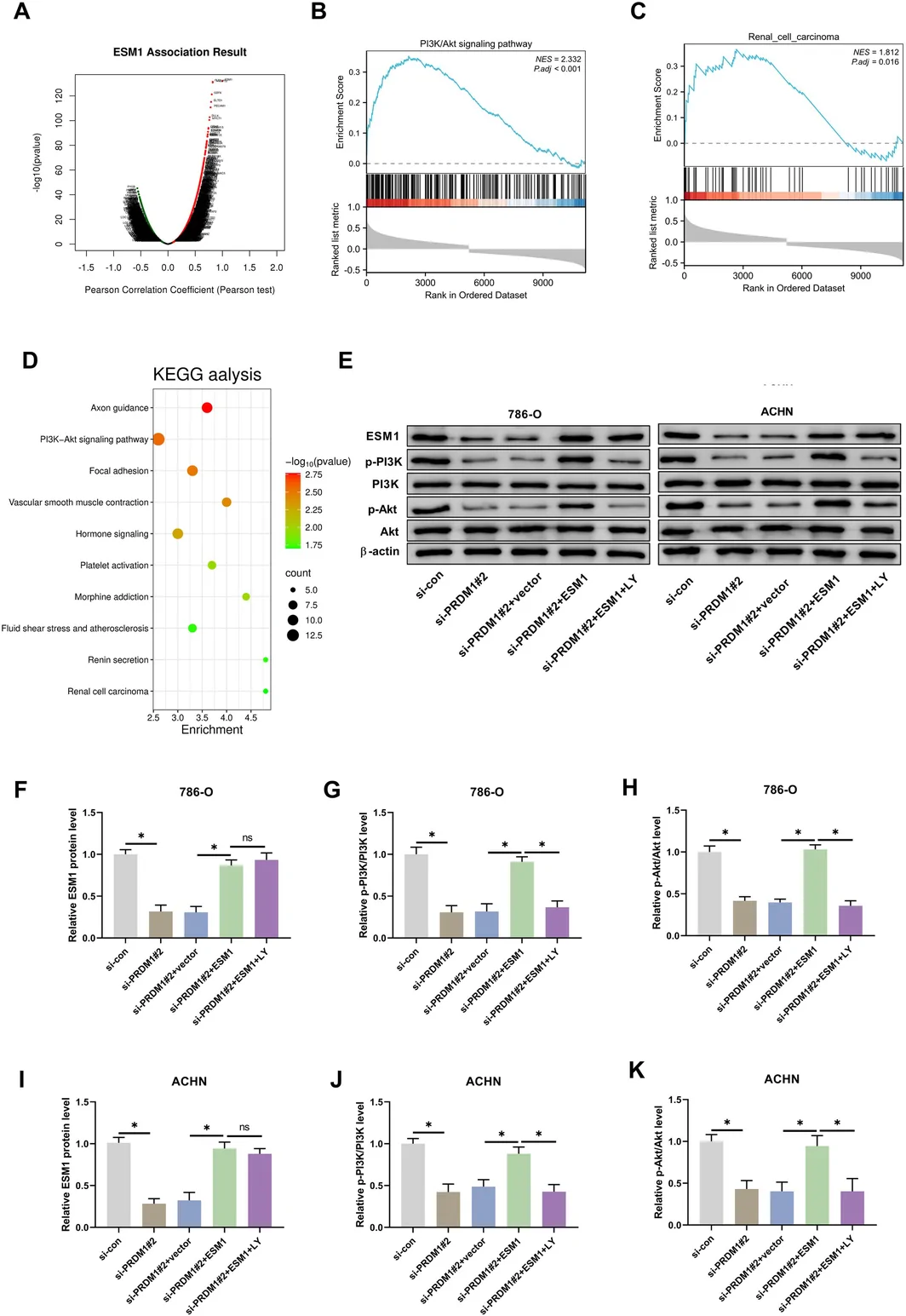
PRDM1 regulates the PI3K/Akt pathway through mediation of ESM1 expression in RCC cells. (A) Linkedomics for the analysis of ESM1 co‐expressed genes in RCC. (B, C) GSEA results of ESM1 co‐expressed genes. (D) KEGG analysis of ESM1 co‐expressed genes. (E–K) Western blot showed that ESM1 mediated the inhibitory effect of si‐PRDM1 on PI3K/Akt signaling. *p < 0.05 between two groups.

To explore the downstream pathways influenced by ESM1 in RCC, ESM1 co‐expressed genes in RCC were analyzed using LinkeDomics (Figure 5A). GSEA indicated that ESM1 was closely associated with PI3K/Akt signaling in RCC (Figure 5B,C). In addition, KEGG analysis showed that genes linked to ESM1 were implicated in PI3K/Akt signaling (Figure 5D). pcDNA/ESM1 was transfected into RCC cell lines to elevate the expression of ESM1 against si‐PRDM1#2 transfection (Figure 5E,F,I). In addition, p‐PI3K/PI3K and p‐Akt/Akt levels were significantly decreased in si‐PRDM1#2‐transfected cells, which was reversed by ESM1 overexpression. Moreover, the inductive effect of ESM1 overexpression on PI3K/Akt signaling was attenuated by LY294002, a PI3K inhibitor (Figure 5E–K).

### 3.6 ESM1 Overexpression Attenuated the Effect of PRDM1 Knockdown on Ferroptosis Through PI3K/Akt Signaling in RCC Cells

**FIGURE 6:**
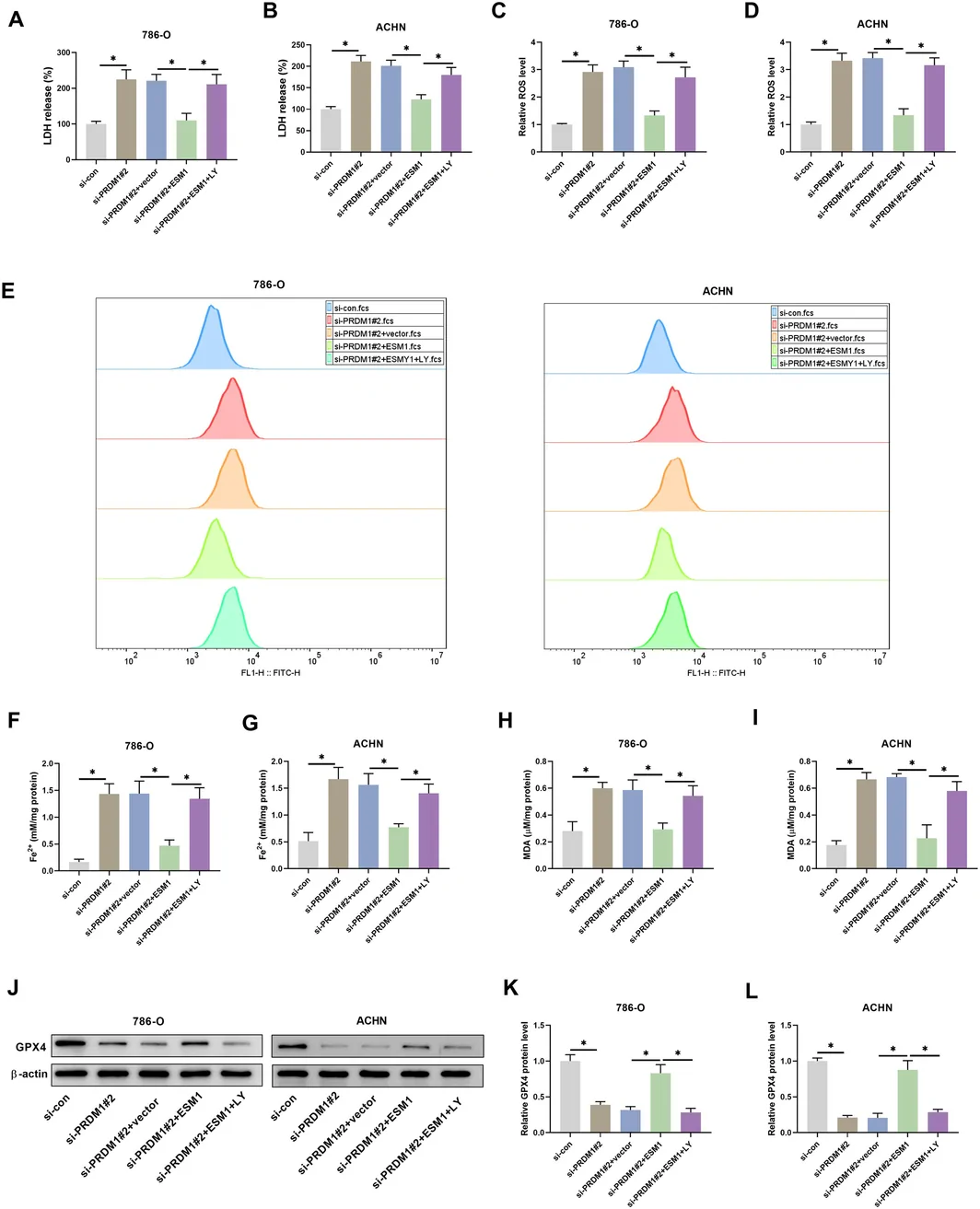
ESM1 overexpression attenuated the effect of PRDM1 knockdown on ferroptosis through PI3K/Akt signaling in RCC cells. RCC cells were transfected with si‐con, si‐PRDM1#2, si‐PRDM1#2 + vector or si‐PRDM1#2 + ESM1 prior to LY294002 (10 μM) treatment. (A, B) Changes in LDH levels. (C–E) Flow cytometry for ROS production. (F–I) Changes in Fe^2+^ level and MDA content. (J–L) Western blot analysis for determination of GPX4 expression. *p < 0.05 between two groups.

To explore whether ESM1 and PI3K/Akt pathways are involved in the PRDM1‐mediated regulation of ferroptosis, RCC cells were transfected with si‐con, si‐PRDM1#2, si‐PRDM1#2 + vector, or si‐PRDM1#2 + ESM1 prior to LY294002 treatment. As shown in Figure 6A,B, the si‐PRDM1#2‐caused increase in LDH levels was mitigated by ESM1 overexpression and was reversed by LY294002. In addition, ESM1 overexpression suppressed ROS production, Fe^2+^ levels, and MDA content in si‐PRDM1#2‐transfected cells (Figure 6C–I). Furthermore, the decreased GPX4 expression levels in si‐PRDM1#2 transfected cells were reversed by ESM1 overexpression; however, the inhibition of PI3K/Akt signaling by LY294002 exhibited the opposite effects against ESM1 overexpression in RCC cells (Figure 6J–L).

### 3.7 ESM1 Overexpression Reversed the Effect of PRDM1 Knockdown on Sunitinib Sensitivity Through PI3K/Akt Signaling in RCC Cells

**FIGURE 7:**
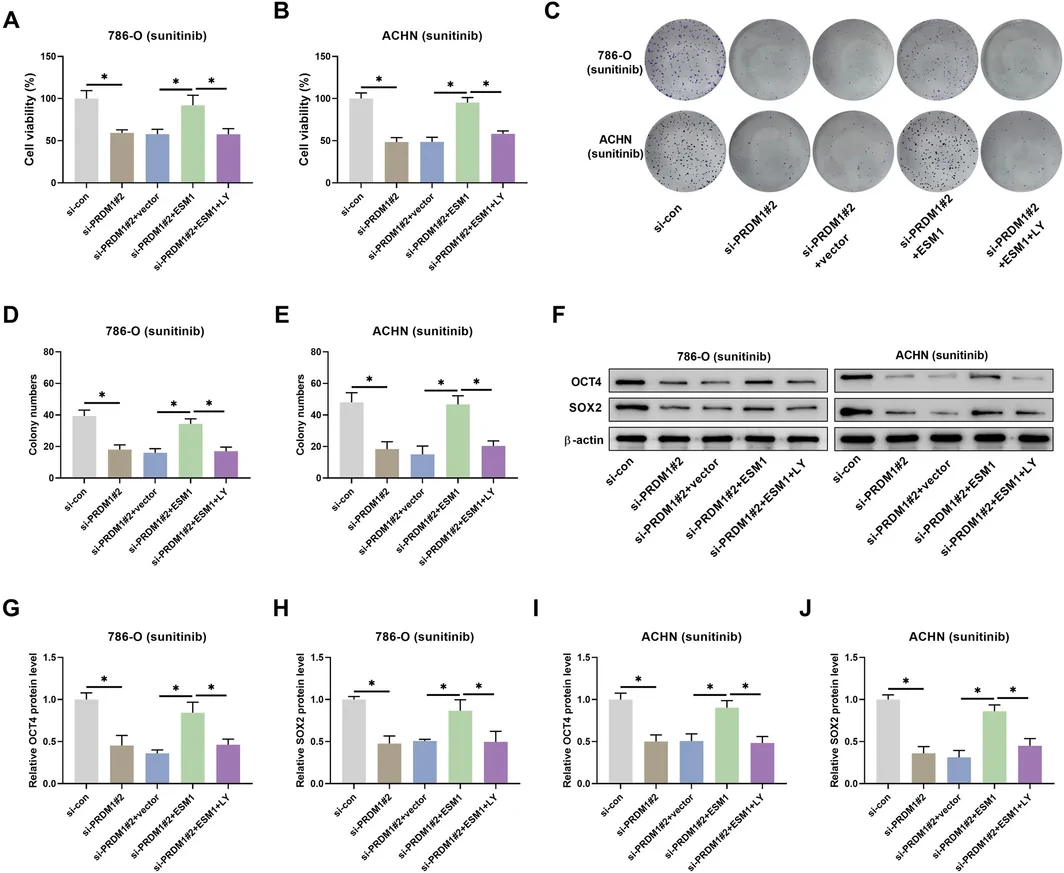
ESM1 overexpression attenuated the effect of PRDM1 knockdown on sunitinib sensitivity through PI3K/Akt signaling in RCC cells. Sunitinib‐treated RCC cells were transfected with si‐con, si‐PRDM1#2, si‐PRDM1#2 + vector or si‐PRDM1#2 + ESM1 prior to LY294002 (10 μM) treatment. (A, B) CCK‐8 assay for detecting cell viability. (C–E) Colony formation assay for detecting cell proliferation ability. (F–J) Western blot for detecting the expression levels of stemness‐associated markers OCT4 and SOX2. *p < 0.05 between two groups.

To determine whether the ESM1 and PI3K/Akt pathways were associated with PRDM1‐mediated regulation of sunitinib sensitivity, sunitinib‐treated RCC cells were transfected with si‐con, si‐PRDM1#2, si‐PRDM1#2 + vector, or si‐PRDM1#2 + ESM1 prior to LY294002 treatment. As indicated in Figure 7A,B, ESM1 overexpression increased the si‐PRDM1#2‐caused reduction in the viability of RCC cells, whereas LY294002 reversed this increase in cell viability. In addition, the si‐PRDM1#2‐caused reduction in colony number was prevented by ESM1 overexpression (Figure 7C–E). Moreover, ESM1 overexpression elevated the expression of OCT4 and SOX2 in si‐PRDM1#2‐transfected cells, which was inhibited by LY294002 (Figure 7F–J).

### 3.8 Knockdown of PRDM1 Inhibited Tumor Growth In Vivo

**FIGURE 8:**
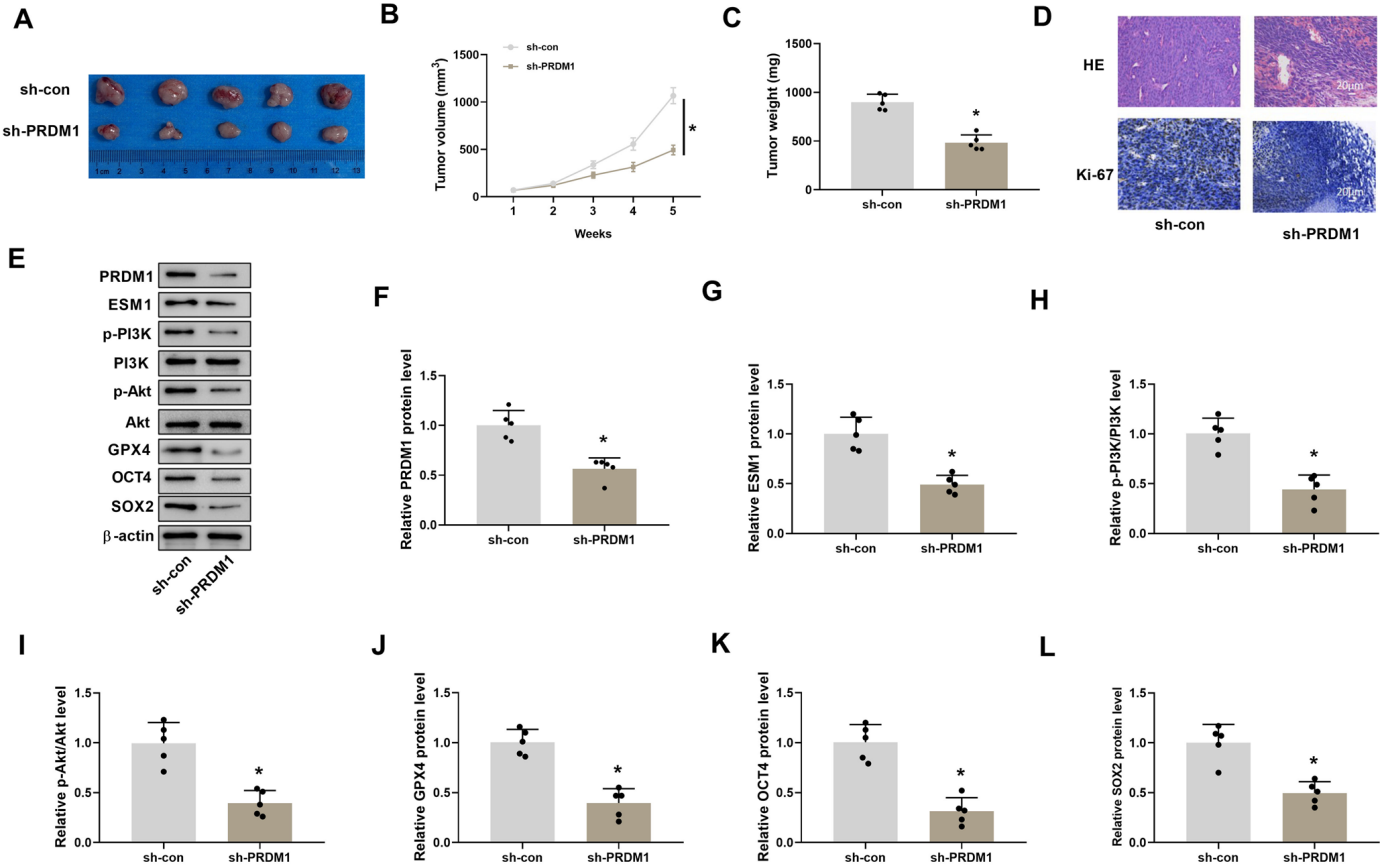
Knockdown of PRDM1 inhibited tumor growth in vivo. (A–C) Knockdown of PRDM1 led to an obvious decrease in tumor volume and weight. (D) HE and IHC staining assays indicated decreased tumor cell proliferation in the sh‐PRDM1 group. (E–L) Western blot showed the decreased expression levels of PRDM1, ESM1, p‐PI3K, p‐Akt, GPX4, OCT4, and SOX2 in the sh‐PRDM1 group. *p < 0.05 versus si‐con control group.

Finally, we investigated the role of PRDM1 in tumor growth in vivo. Our results showed that PRDM1 expression was markedly downregulated in the sh‐PRDM1 group (Figure 8E,F), which led to an obvious decrease in tumor volume and weight (Figure 8A–C). H&E and Ki‐67 staining assays indicated that PRDM1 silencing attenuated tumor tissue growth (Figure 8D). Western blot assays showed that the levels of p‐PI3K/PI3K and p‐Akt/Akt were greatly reduced in the sh‐PRDM1 group, indicating inhibition of the PI3K/Akt signaling pathway (Figure 8E,H,I). In addition, the knockdown of PRDM1 caused a remarkable decrease in the expression levels of GPX4, OCT4, and SOX2 in tumor tissues (Figure 8E,J–L). These results indicated that the knockdown of PRDM1 inhibited tumor growth by increasing ferroptosis and sunitinib sensitivity.

## 4 Discussion

An increasing number of studies have documented the role of PRDM1 in various human cancers, highlighting the role of PRDM1 as a tumor regulator. PRDM1 is frequently inactivated in several types of lymphomas, such as anaplastic large T‐cell lymphoma, Natural Killer cell lymphoma, and diffuse large B‐cell lymphoma [14, 19]. It also serves as a tumor suppressor gene in B‐ and T‐cell lymphomas [14]. Kang et al. [20] reported that PRDM1 has tumor‐suppressive activity in human colon cancer: forced expression of PRDM1 prevents the formation and growth of human colon tumor organoids, and lower expression levels of PRDM1 predict poor survival in patients with colon cancer [21]. PRDM1 depletion in lung cancer cells promotes anoikis resistance and cellular invasion in vitro, as well as promotes lung metastasis in vivo [18]. These findings suggest that PRDM1 exhibits tumor‐suppressive activity in these cancers.

In addition, PRDM1 was found to exert tumor‐promoting effects in other types of cancers. High expression levels of PRDM1 are detected in invasive breast cancer cells, which correlates with metastatic status in patients [22]. PRDM1 triggers breast cancer cell invasion and metastasis formation [22]. Zhou et al. [17] demonstrated that PRDM1 expression is significantly increased in PAAD, and high expression of PRDM1 is associated with poor prognosis in patients with PAAD. In hepatocellular carcinoma, PRDM1 overexpression promotes tumor cell immune evasion [15]. PRDM1 could contribute to cancer progression by promoting stemness of gastric cancer [23]. In line with previous studies, we found that PRDM1 expression was markedly upregulated in RCC tissues and different RCC cell lines. We hypothesized the anti‐cancer or oncogenic role of PRDM1 in different cancers might be associated with the difference in tumor microenvironment.

Ferroptosis is a novel type of programmed cellular death, exceptionally characterized by an iron‐dependent manner [8]. Ferroptosis has garnered substantial interest as an innate tumor suppressor mechanism [24]. Extensive research has proven that ferroptosis participates in a variety of biological processes in the development and progression of tumors [25]. Consequently, targeting ferroptosis may contribute to the destruction of tumor cells and prevention of tumor progression, which has been considered a promising therapeutic technique for tumor treatment. Ma et al. reported that PRDM1 induces ferroptosis in thyroid cancer cells by regulating the deubiquitination of ubiquitin‐specific peptidase 15 (USP15)‐mediated selenium‐binding protein 1 (SELENBP1) [16]. In this study, we found that knockdown of PRDM1 promoted ferroptosis in RCC cells and xenografts. Although chemotherapy benefits patients with advanced RCC, sunitinib resistance leads to poor prognosis [26]. Moreover, ferroptosis is closely associated with chemoresistance in tumors, including RCC [11]. Next, we evaluated the effect of PRDM1 on sunitinib resistance. We found that PRDM1 knockdown enhanced the sensitivity of RCC cells to sunitinib. Cancer stemness has been identified as a key component of tumor development [27]. Recent advances in cancer research have demonstrated that enhancing the stemness of cancer cells may accelerate their proliferation, invasion, metastasis [28], epithelial‐to‐mesenchymal transition (EMT) [29], and chemotherapy resistance [30]. We also found that knockdown of PRDM1 decreased the expression levels of the stemness‐associated markers, OCT4 and SOX2. These results indicate that PRDM1 knockdown promotes ferroptosis and elevates the sensitivity of RCC cells to sunitinib.

ESM1 is pathologically upregulated in multiple cancer types and is closely involved in the progression of multiple cancers, such as angiogenesis, drug resistance, proliferation, migration, invasion, and apoptosis escape [31]. ESM1 is also markedly overexpressed in RCC and serves as a promising prognostic biomarker in RCC [32]. However, its exact role in RCC has not been reported. In the present study, we found that PRDM1 binds directly to ESM1 and regulates its transcription. ESM1 overexpression reverses the effects of si‐PRDM1 on ferroptosis and sunitinib sensitivity in RCC cells. Previous reports have suggested PRDM1 could regulate immune evasion in thyroid or hepatocellular carcinoma through modulating USP15‐mediated SELENBP1 deubiquitination or USP22‐mediated SPI1 deubiquitination [15, 16]. The current study explored a novel target of PRDM1 in RCC. To the best of our knowledge, our research is the first to establish that the PRDM1/ESM1 regulatory axis regulates ferroptosis and sunitinib sensitivity in RCC cells. Previous studies indicated that ESM1 could regulate diverse molecular signaling pathways including the PI3K/Akt pathway, thereby affecting the development and progression of cancer [31, 33]. To further explore the biological role of ESM1 in RCC, we investigated the effect of ESM1 on PI3K/Akt signaling, which is a critical signaling pathway overactivated in tumor progression and chemoresistance [34]. Our results showed that ESM1 was closely associated with PI3K/Akt signaling in RCC, and ESM1 overexpression reversed the effects of PRDM1 knockdown on ferroptosis and sunitinib sensitivity through PI3K/Akt signaling in RCC cells. These observations suggested that PRDM1 regulates ferroptosis and resistance through the ESM1‐mediated PI3K/Akt pathway in RCC.

However, there are some limitations in the current study. Firstly, many factors are involved in ferroptosis, while this study just investigates the effects on GPX4 in this process. More key factors should be analyzed for ferroptosis assessment in the future. Secondly, our study confirmed PRDM1 could regulate the PI3K/Akt signaling, while PRDM1 was suggested as a downstream of the PI3K/Akt pathway [35]. The potential feedback regulation between PRDM1 and PI3K/Akt signaling should be analyzed in RCC in the future. Thirdly, the promoter truncation or site‐directed mutagenesis is absent, leaving PRDM1's direct transcriptional control of ESM1 unproven. Moreover, more downstream pathways should be explored to better understand the anti‐cancer mechanism of PRDM1. Targeting PRDM1 and potential effectors could provide a promising strategy for anti‐cancer therapy to RCC.

In conclusion, our study demonstrates that PRDM1 silencing might promote ferroptosis and sunitinib sensitivity in RCC possibly via the ESM1/PI3K/Akt axis. These findings provide new insights into the role of PRDM1 in tumor research.

## Funding

This work was supported by grants from the National Natural Science Foundation of China (82273106).

## Conflicts of Interest

The authors declare no conflicts of interest.

## Supporting information


**Figure S1:** Addition of ferroptosis inhibitor attenuated the effects of PRDM1 knockdown on ferroptosis in RCC cell lines. 786‐O and ACHN cells were transfected with si‐con or si‐PRDM1#2 and treated with a ferroptosis inhibitor Fer‐1. (A, B) LDH, (C, D) Fe^2+^, (E–H) ROS, (I, J) MDA, and (K–N) GPX4 protein levels were detected in cells. *p < 0.05 between two groups.

## Data Availability

The datasets used and/or analyzed during the current study are available from the corresponding author on reasonable request.

## References

[1] S. A. Padala , A. Barsouk , K. C. Thandra , et al., “Epidemiology of Renal Cell Carcinoma,” World Journal of Oncology 11, no. 3 (2020): 79–87. 32494314 10.14740/wjon1279 PMC7239575

[2] R. L. Siegel , K. D. Miller , N. S. Wagle , and A. Jemal , “Cancer Statistics, 2023,” CA: A Cancer Journal for Clinicians 73, no. 1 (2023): 17–48. 36633525 10.3322/caac.21763

[3] L. Meng , K. A. Collier , P. Wang , et al., “Emerging Immunotherapy Approaches for Advanced Clear Cell Renal Cell Carcinoma,” Cells 13, no. 1 (2023): 34. 38201238 10.3390/cells13010034 PMC10777977

[4] K. Wang , Y. Ding , Y. Liu , et al., “CPA4 as a Biomarker Promotes the Proliferation, Migration and Metastasis of Clear Cell Renal Cell Carcinoma Cells,” Journal of Cellular and Molecular Medicine 28, no. 7 (2024): e18165. 38494845 10.1111/jcmm.18165 PMC10945090

[5] M. B. Atkins and N. M. Tannir , “Current and Emerging Therapies for First‐Line Treatment of Metastatic Clear Cell Renal Cell Carcinoma,” Cancer Treatment Reviews 70 (2018): 127–137. 30173085 10.1016/j.ctrv.2018.07.009

[6] A. M. Molina , X. Lin , B. Korytowsky , et al., “Sunitinib Objective Response in Metastatic Renal Cell Carcinoma: Analysis of 1059 Patients Treated on Clinical Trials,” European Journal of Cancer 50, no. 2 (2014): 351–358. 24051327 10.1016/j.ejca.2013.08.021

[7] J. Jin , Y. Xie , J. S. Zhang , et al., “Sunitinib Resistance in Renal Cell Carcinoma: From Molecular Mechanisms to Predictive Biomarkers,” Drug Resistance Updates 67 (2023): 100929. 36739809 10.1016/j.drup.2023.100929

[8] X. Jiang , B. R. Stockwell , and M. Conrad , “Ferroptosis: Mechanisms, Biology and Role in Disease,” Nature Reviews. Molecular Cell Biology 22, no. 4 (2021): 266–282. 33495651 10.1038/s41580-020-00324-8 PMC8142022

[9] T. Xu , H. Liu , N. Ling , et al., “OTUD3‐Mediated Stabilization of SLC7A11 Drives Sunitinib Resistance by Suppressing Ferroptosis in Clear Cell Renal Cell Carcinoma,” Cancer Letters 632 (2025): 217942. 40716486 10.1016/j.canlet.2025.217942

[10] B. Xu , W. J. Zhu , Y. J. Peng , and S. D. Cheng , “Curcumin Reverses the Sunitinib Resistance in Clear Cell Renal Cell Carcinoma (ccRCC) Through the Induction of Ferroptosis via the ADAMTS18 Gene,” Translational Cancer Research 10, no. 7 (2021): 3158–3167. 35116623 10.21037/tcr-21-227 PMC8797884

[11] X. Tian , J. Liu , C. Yi , X. You , and C. Yuan , “Hsa_circ_0072732 Enhances Sunitinib Resistance of Renal Cell Carcinoma by Inhibiting Ferroptosis,” Discover Oncology 15, no. 1 (2024): 700. 39580569 10.1007/s12672-024-01580-2 PMC11585529

[12] C. Yu , J. Li , W. Kuang , S. Ni , Y. Cao , and Y. Duan , “PRDM1 Promotes Nucleus Pulposus Cell Pyroptosis Leading to Intervertebral Disc Degeneration via Activating CASP1 Transcription,” Cell Biology and Toxicology 40, no. 1 (2024): 89. 39432156 10.1007/s10565-024-09932-y PMC11493826

[13] T. Yoshikawa , Z. Wu , S. Inoue , et al., “Genetic Ablation of PRDM1 in Antitumor T Cells Enhances Therapeutic Efficacy of Adoptive Immunotherapy,” Blood 139, no. 14 (2022): 2156–2172. 34861037 10.1182/blood.2021012714

[14] M. Boi , E. Zucca , G. Inghirami , and F. Bertoni , “PRDM1/BLIMP1: A Tumor Suppressor Gene in B and T Cell Lymphomas,” Leukemia & Lymphoma 56, no. 5 (2015): 1223–1228. 25115512 10.3109/10428194.2014.953155

[15] Q. Li , L. Zhang , W. You , et al., “PRDM1/BLIMP1 Induces Cancer Immune Evasion by Modulating the USP22‐SPI1‐PD‐L1 Axis in Hepatocellular Carcinoma Cells,” Nature Communications 13, no. 1 (2022): 7677. 10.1038/s41467-022-35469-x PMC9744896 36509766

[16] J. Ma , Z. Li , J. Xu , et al., “PRDM1 Promotes the Ferroptosis and Immune Escape of Thyroid Cancer by Regulating USP15‐Mediated SELENBP1 Deubiquitination,” Journal of Endocrinological Investigation 47, no. 12 (2024): 2981–2997. 39014173 10.1007/s40618-024-02385-4

[17] B. Zhou , J. Zhang , H. Zhu , and S. Wu , “A Potential Prognostic Marker PRDM1 in Pancreatic Adenocarcinoma,” Journal of Oncology 2022 (2022): 1934381. 35607327 10.1155/2022/1934381 PMC9123419

[18] Z. Zhu , H. Wang , Y. Wei , F. Meng , Z. Liu , and Z. Zhang , “Downregulation of PRDM1 Promotes Cellular Invasion and Lung Cancer Metastasis,” Tumor Biology 39, no. 4 (2017): 1010428317695929. 28378641 10.1177/1010428317695929

[19] C. Kucuk , J. Iqbal , X. Hu , et al., “PRDM1 Is a Tumor Suppressor Gene in Natural Killer Cell Malignancies,” Proceedings of the National Academy of Sciences of the United States of America 108, no. 50 (2011): 20119–20124. 22143801 10.1073/pnas.1115128108 PMC3250125

[20] H. B. Kang , H. R. Lee , J. da Jee , et al., “PRDM1, a Tumor‐Suppressor Gene, Is Induced by Genkwadaphnin in Human Colon Cancer SW620 Cells,” Journal of Cellular Biochemistry 117, no. 1 (2016): 172–179. 26096175 10.1002/jcb.25262

[21] C. Liu , C. E. Banister , C. C. Weige , et al., “PRDM1 Silences Stem Cell‐Related Genes and Inhibits Proliferation of Human Colon Tumor Organoids,” Proceedings of the National Academy of Sciences of the United States of America 115, no. 22 (2018): E5066–E5075. 29760071 10.1073/pnas.1802902115 PMC5984534

[22] M. Sciortino , M. D. P. Camacho‐Leal , F. Orso , et al., “Dysregulation of Blimp1 Transcriptional Repressor Unleashes p130Cas/ErbB2 Breast Cancer Invasion,” Scientific Reports 7, no. 1 (2017): 1145. 28442738 10.1038/s41598-017-01332-z PMC5430666

[23] H. Qin , M. Yuan , Y. Yuan , X. Liu , and S. Yi , “PRDM1 Promotes the Stemness of Gastric Cancer Cells by Enhancing the Transactivation of Myc,” Translational Oncology 59 (2025): 102443. 40532656 10.1016/j.tranon.2025.102443 PMC12212116

[24] B. R. Stockwell , “Ferroptosis Turns 10: Emerging Mechanisms, Physiological Functions, and Therapeutic Applications,” Cell 185, no. 14 (2022): 2401–2421. 35803244 10.1016/j.cell.2022.06.003 PMC9273022

[25] Q. Zhou , Y. Meng , D. Li , et al., “Ferroptosis in Cancer: From Molecular Mechanisms to Therapeutic Strategies,” Signal Transduction and Targeted Therapy 9, no. 1 (2024): 55. 38453898 10.1038/s41392-024-01769-5 PMC10920854

[26] Y. He , Y. Luo , L. Huang , et al., “New Frontiers Against Sorafenib Resistance in Renal Cell Carcinoma: From Molecular Mechanisms to Predictive Biomarkers,” Pharmacological Research 170 (2021): 105732. 34139345 10.1016/j.phrs.2021.105732

[27] J. J. Loh and S. Ma , “Hallmarks of Cancer Stemness,” Cell Stem Cell 31, no. 5 (2024): 617–639. 38701757 10.1016/j.stem.2024.04.004

[28] M. Peiris‐Pages , U. E. Martinez‐Outschoorn , R. G. Pestell , F. Sotgia , and M. P. Lisanti , “Cancer Stem Cell Metabolism,” Breast Cancer Research 18, no. 1 (2016): 55. 27220421 10.1186/s13058-016-0712-6 PMC4879746

[29] Q. Tang , J. Chen , Z. Di , et al., “TM4SF1 Promotes EMT and Cancer Stemness via the Wnt/Beta‐Catenin/SOX2 Pathway in Colorectal Cancer,” Journal of Experimental & Clinical Cancer Research 39, no. 1 (2020): 232. 33153498 10.1186/s13046-020-01690-z PMC7643364

[30] T. Qin , B. Li , X. Feng , et al., “Abnormally Elevated USP37 Expression in Breast Cancer Stem Cells Regulates Stemness, Epithelial‐Mesenchymal Transition and Cisplatin Sensitivity,” Journal of Experimental & Clinical Cancer Research 37, no. 1 (2018): 287. 30482232 10.1186/s13046-018-0934-9 PMC6258492

[31] Y. K. Li , T. Zeng , Y. Guan , et al., “Validation of ESM1 Related to Ovarian Cancer and the Biological Function and Prognostic Significance,” International Journal of Biological Sciences 19, no. 1 (2023): 258–280. 36594088 10.7150/ijbs.66839 PMC9760436

[32] K. Bocu , A. F. Batur , Z. E. Celik , et al., “Prognostic Role of the Endothelial Cell‐Specific Molecule‐1 Histopathologic Expression in Renal Cell Cancer,” Urologic Oncology 41, no. 6 (2023): 297.e1–297.e9. 10.1016/j.urolonc.2023.03.008 37127479

[33] L. Yang , Z. Dong , S. Li , and T. Chen , “ESM1 Promotes Angiogenesis in Colorectal Cancer by Activating PI3K/Akt/mTOR Pathway, Thus Accelerating Tumor Progression,” Aging (Albany NY) 15, no. 8 (2023): 2920–2936. 37100467 10.18632/aging.204559 PMC10188330

[34] A. Glaviano , A. S. C. Foo , H. Y. Lam , et al., “PI3K/AKT/mTOR Signaling Transduction Pathway and Targeted Therapies in Cancer,” Molecular Cancer 22, no. 1 (2023): 138. 37596643 10.1186/s12943-023-01827-6 PMC10436543

[35] C. E. Faliti , M. Mesina , J. Choi , et al., “Interleukin‐2‐Secreting T Helper Cells Promote Extra‐Follicular B Cell Maturation via Intrinsic Regulation of a B Cell mTOR‐AKT‐Blimp‐1 Axis,” Immunity 57, no. 12 (2024): 2772–2789.e8. 39612915 10.1016/j.immuni.2024.11.006 PMC11675998

