# Supplementary figures and images for: PRDM1 Knockdown Promotes Ferroptosis and Sunitinib Sensitivity by Modulating the PI3K/Akt Signaling Through Inhibition of ESM1 Transcription in Renal Cell Carcinoma
Source: Kaohsiung J Med Sci. 2026 Feb 16:e70187. Online ahead of print. doi: 10.1002/kjm2.70187 (PMC13399922; doi:10.1002/kjm2.70187)

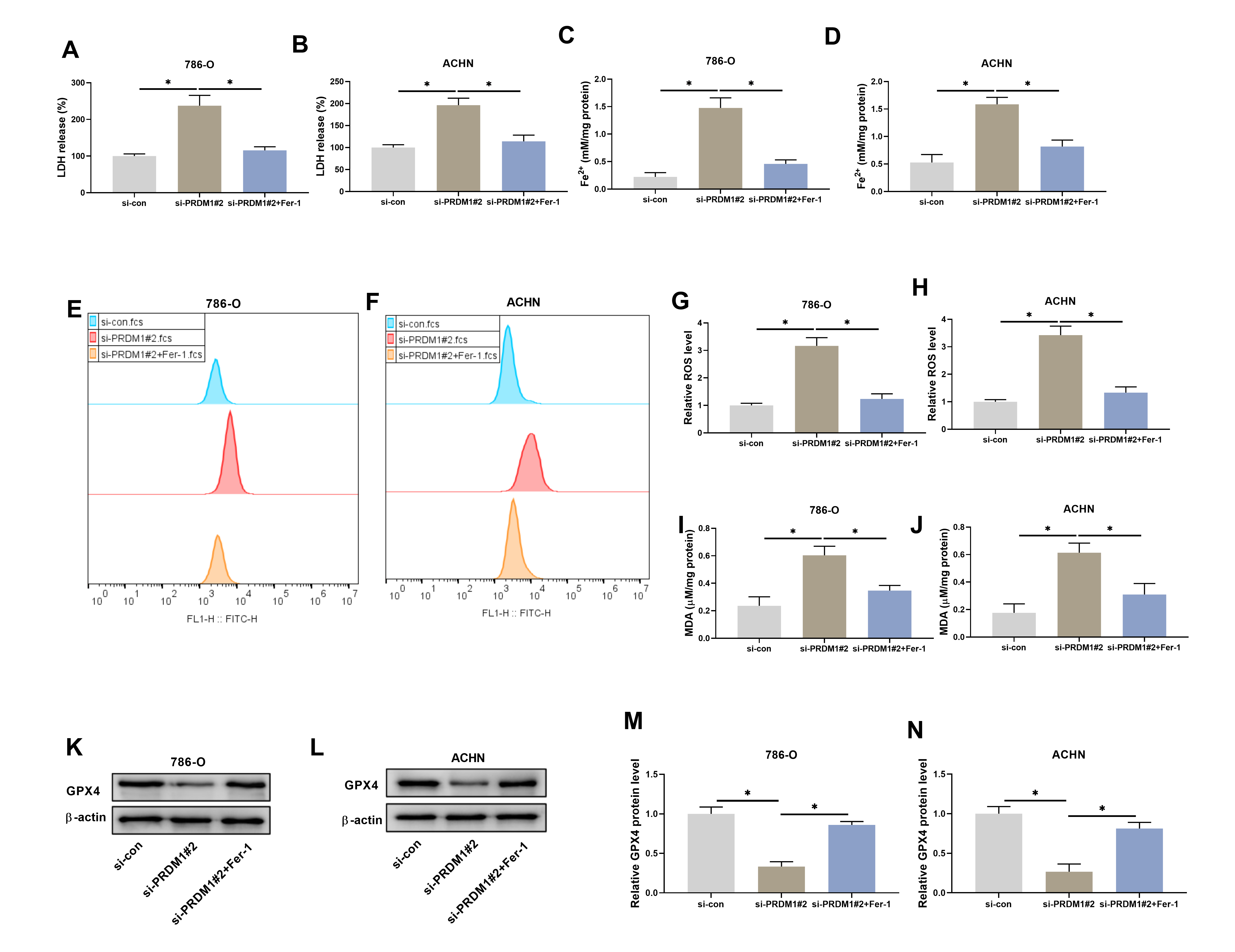

Supplement: Supplementary file 1 — Figure S1: Addition of ferroptosis inhibitor attenuated the effects of PRDM1 knockdown on ferroptosis in RCC cell lines. 786‐O and ACHN cells were transfected with si‐con or si‐PRDM1#2 and treated with a ferroptosis inhibitor Fer‐1. (A, B) LDH, (C, D) Fe2+, (E–H) ROS, (I, J) MDA, and (K–N) GPX4 protein levels were detected in cells. *p < 0.05 between two groups. [file KJM2-9999-e70187-s001.tif]
